# Influences of Air-Voids on the Performance of 3D Printing Cementitious Materials

**DOI:** 10.3390/ma14164438

**Published:** 2021-08-08

**Authors:** Yujun Che, Shengwen Tang, Huashan Yang, Weiwei Li, Mengyuan Shi

**Affiliations:** 1 School of Materials and Architecture Engineering, Guizhou Normal University, Guiyang 550025, China; 201602003@gznu.edu.cn (Y.C.); 201510003@gznu.edu.cn (H.Y.); ghtliweiwei@163.com (W.L.); s758982737@163.com (M.S.); 2 State Key Laboratory of Water Resources and Hydropower Engineering Science, Wuhan University, Wuhan 430072, China; 3 State Key Laboratory of Building Safety and Built Environment, Beijing 100013, China; 4 National Engineering Research Center of Building Technology, Beijing 100053, China; 5 State Key Laboratory of Green Building Materials, China Building Materials Academy, Beijing 100024, China; 6 Suzhou Institute of Wuhan University, Suzhou 215123, China

**Keywords:** 3D printing cementitious materials, cementitious materials, air-void structure, anti-foaming agent, strength development

## Abstract

This paper focuses on inspecting the influences of anti-foaming agent (AFA) on the performance of 3D printing cementitious materials (3DPC). The mini-slump, spreading diameter, yield stress, and strength of 3DPC were evaluated. Additionally, the air-void content, air-void morphology, and air-void size distribution of mortar with and without 0.05% AFA were assessed through image analysis. The mechanical performance and air-void structure of 3D printed samples were also investigated and compared to that of conventionally mould cast samples. Test results show that an optimal AFA content enables 3DPC to achieve favorable workability and mechanical performance. The addition of AFA exhibits lower air-void content in 3DPC than that of the sample without the AFA addition. This reduction in air-void content is further strengthened by the results of strength analysis. Electron microscope analysis shows that the use of AFA results in the suppressed formation of large air-voids during the process of fresh 3DPC. Moreover, the air-void morphology substantially influenced the mechanical performance of hardened 3DPC.

## 1. Introduction

When considering 3D printing concrete technology, an extrusion-based additive construction technique [1] could be used to build complex construction structures layer-by-layer due to its rapid prototyping. This innovative construction process has recently seen a rapid development for civil engineering structures due to its distinct advantages, such as complex manufacturing, increased efficiency, construction automation, and environmental protection [2,3,4,5,6,7]. Contour Crafting developed at the University of Southern California and Concrete Printing developed at Loughborough University have been applied to the production of complex concrete components [8,9]. There is also significant potential to construct special hydraulic structures using this technology. The viability of 3D printing concrete technology for hydraulic buildings with irregular shapes and complex structures, such as overflow weir surfaces, diversion tunnel entrances and exits, and hydropower station draft tubes, is now being explored.

While the advantages of 3D printing cementitious materials (3DPC) have been studied by many researchers [10,11,12,13,14], advanced 3DPC is still under development with various restrictions, such as unreliable manufacturing processes, weak joints, low mechanical strength, and anisotropic performance in recent literatures [15,16,17]. One such obstacle is unavoidable weak joints between printed layers compared with mould cast cementitious materials, which are the weakest links in 3DPC [18,19]. Generally, the mechanical performance and durability of hardened 3DPC are controlled by the weakest links [20,21,22], as potential flaws can be created between printed layers [23]. The layer-by-layer deposition process of 3DPC commonly introduces numerous interfaces that are primarily caused by air-void attendance between the subsequent filaments [1]. The excessive air-void content has an adverse influence on the performance of 3DPC. Fonseca et al. [24] reported that an entrained air-void directly influences both the workability and durability of cementitious materials. Rahul et al. [25] found that the porosity at the interface between the horizontal and vertical layers was higher than that of mould cast concrete. The air-void between layers is also likely to weaken the bond strength between filaments, thus influencing the hardened performance of 3DPC [26]. Microscopic observation has also revealed that the air-voids and other defects were at the printed concrete layers [27]. A lack of compaction procedure in layer-by-layer deposition processes results in the presence of excessive air-voids. As a result, 3DPC exhibits a more extensive air-void structure, which leads to the decrease of bond strength between the layers [27]. Therefore, the air-voids are significantly crucial in the context of the mechanical performance of 3DPC. A comprehensive understanding of the air-void structure is required to enhance the performance of a particular mix.

Air-void content has an adverse influence on the strength and durability of hardened cementitious materials [28,29]. For instance, the compressive strength of high-strength concrete reduces about 5% for each 1% increase in air content [30]. In addition, air-void size distribution strongly affects the performance of cementitious materials [31,32,33,34,35,36,37]. Although many studies have investigated air-voids on the performance of mould cast cementitious materials, few of them have focused on 3DPC. Existing literature suggests that the 3D printing process affects air-void parameters of 3DPC, such as the air-void content, air-void morphology, and air-void size distribution [38]. Thus, it remains a significant challenge to control the air-void structure. There has been no comprehensive study that has been published investigating the air-void structure from a sub-micro perspective to the best of the authors’ knowledge.

One method for modifying cementitious materials is incorporating anti-foaming agent (AFA)into materials, which can effectively decrease the air-void content [39,40]. Excessive air-voids in 3DPC can also be partly reduced by the addition of AFA. Therefore, a comprehensive and in-depth investigation of AFA on the strength development of 3DPC is of great importance. Moreover, the performance of cementitious materials containing numerous air-void is influenced by the spatial distribution of the air-void [41]. Therefore, the investigation of the influences of AFA on the air-void distribution in 3DPC is required. Regarding air-voids, not only is the content and size distribution important, but their morphology is important as well. The air-void morphology is also an important parameter that also significantly influences the performance of 3DPC.

In recognition of the importance of air-void, the present work focuses on the mini-slump, spreading diameter, and yield stress of fresh 3DPC as they are influenced by AFA dosages. Furthermore, the influences of the air-void parameters, such as air-void content, air-void morphology, and air-void size distribution, on the strength development of hardened 3DPC are investigated. The compressive strength and air-void structure of the mould cast samples are also investigated and compared to that of the counterpart 3D printed samples. The research outcome is a detailed understanding of the potential application of 3DPC containing AFA for the construction of special structures where customization is important. The results can be applied to modify the air-void structure of 3DPC, thus improving the performance of the materials.

## 2. Materials and Methods

**Figure 1:**
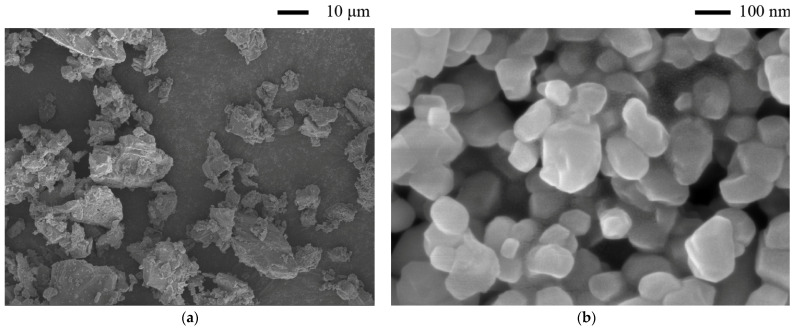
SEM of LS and NC, (**a**) LS, (**b**) NC.

**Figure 2:**
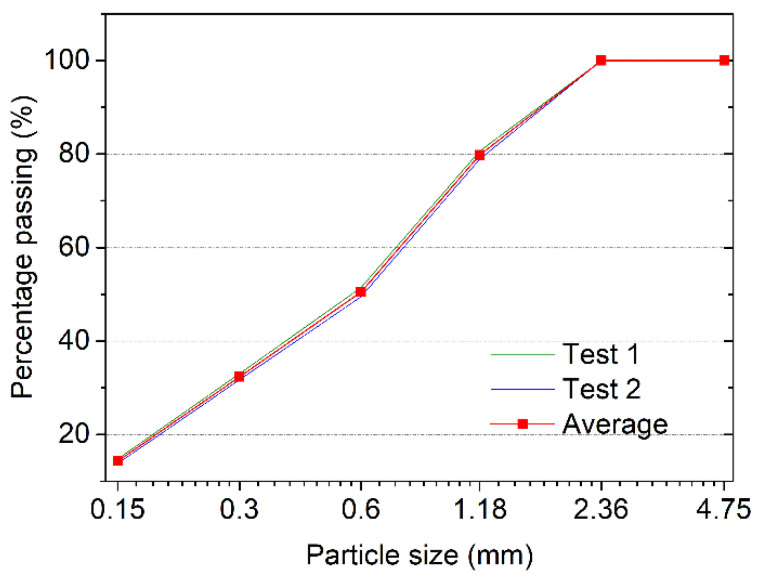
Particle size distribution of quartz sand.

All mixtures were produced with Portland cement (PC) (P·I 42.5, China United Cement Corporation, Qufu, China), limestone powder (LS), Nano-CaCO_3_ (NC), fine aggregate, tap water, AFA, high-range water-reducing admixture (HRWRA), and hydroxypropyl methylcellulose (HPMC). All of them (excluding tap water) are commercial products. Table 1 gives the chemical composition of the Portland cement obtained by X-ray fluorescence spectrometry (XRF). Portland cement and LS with an average particle diameter of 33.3 μm were used as binders. LS was added to the cement at 10% of the weight of binders. NC with a 40 nm average particle diameter was added at a ratio of 1% per the mass of the binders. Figure 1a,b show scanning electron microscope (SEM) images of the LS and NC, illustrating the particle size and morphology of two materials. The addition of LS and NC in cementitious materials significantly increases the structural build-rate [42,43], which is detrimental in the layer-by-layer deposition of 3DPC. To evaluate the influences of air-voids on the performance of 3DPC, an AFA was added at ratios of 0, 0.05, 0.1, 0.5, and 1.0% per the mass of the binders. HRWRA and HPMC were used to modify the flowability and buildability of fresh state 3DPC, respectively. Fine aggregates were quartz sand with a particle size in the range of 0.15–2.36 mm, as shown in Figure 2.

The investigation involves two types of samples: 3D printed mortar and mould cast mortar. The mix proportions reported herein are given in Table 2. The mixtures are labeled with respect to the dosage of AFA. For instance, AFA005 represents a sample with 0.05% AFA. All mortars were mixed in a planetary-style mixer using a water to binder ratio of 0.40, and a binder to sand ratio of 0.50. PC, LS, NC, and fine aggregate were mixed at a slow speed for about 60 s. AFA, HRWRA, and HPMC dissolved in water was added thereafter and mixed at a slow speed for another 60 s. The mixer was then stopped for 90 s to homogenize the mortar manually. This was followed by mixing at a high speed for 60 s. Afterward, the mixture was delivered to the 3D printing system to prepare the samples for strength and air-void structure investigation.

Fluidity is a crucial factor to consider, as it controls the pumpability and extrudability of the fresh 3DPC [44]. The fluidity of the samples was measured by the drop table test and mini-slump in this work. The mini-slump mould dimension was 150 mm in height, 100 mm in bottom diameter, and 50 mm in top diameter.

Yield stress is essential for fresh 3DPC since it affects the stiffness of materials during the 3D printing process. The evolution of yield stress with time is an indicator of the structural build-up of cementitious materials. In this work, the yield stress of the samples was evaluated by a Vicat apparatus. The yield stress of the samples can be calculated by Equation (1) [45]:

τ_0_ = 3/(2πRh) (1)

where τ_0_ is the yield stress of the sample, R is the radius of the Vicat plunger, and its value is 5mm. h is the penetration depth of the Vicat plunger in the sample. Each sample was tested every 15 min until 90 min.

**Figure 3:**
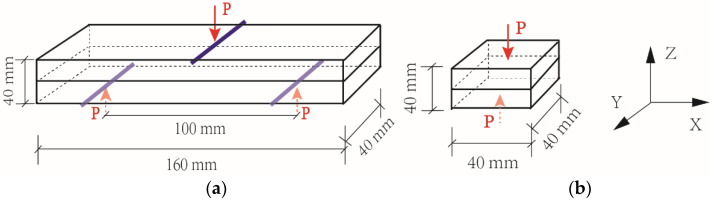
Loading in the Z direction. (**a**) Flexural load in printed objects. (**b**) Compressive load in printed objects.

A gantry concrete 3D printing system (HC-3DPRT/D, Jianyanhua testing (Hangzhou) Technology Co., Ltd., Hangzhou, China) was used to print 4-layer samples with dimensions of roughly 40 mm × 40 mm × 160 mm in this study. The printing speed and extrusion rate were set as 0.19 L/min and 60 cm/min in this paper. The 3D printed samples were cured in a moist cabinet with a temperature of 20 ± 2 °C until testing. The printed samples were then saw-cut into a prism with a dimension of 40 mm × 40 mm × 160 mm for the flexural and compressive strength measurements. The flexural strength and compressive strength of samples were tested according to GB/T 17671-1999 using a loading rate of 50 N/s and 2400 N/s, respectively. Figure 3a,b show the loading direction of the flexural strength and the compressive strength of 3DPC. The compressive strength of the mould cast prisms with a dimension of 40 mm × 40 mm × 160 mm was also tested at 7 and 28 days. The ratio of compressive strength of 3D printed mortar and mould cast mortar, f_P_/f_C_, could then be calculated.

The electron microscope samples were sawed from the 3D printed mortar and the mould cast mortar that had been cured for 28 days. The samples were then cut to expose a fresh surface and were polished using silicon carbide paper. Afterward, electron microscopy images of the samples were captured at ×120 magnification. A modified watershed image processing technique was then applied to segment the air-voids whose diameter was larger than 0.1 mm. Additional investigation will be needed to study the influences of air-voids smaller than 0.1 mm on the performance of 3DPC, which cannot be covered in this paper. There were 32 fields in each sample that were randomly chosen to calculate the air-void content, air-void size distribution, and air-void aspect ratio. Air-void morphology was quantified using its aspect ratio, a practical and straightforward approach for identifying its sphericity. The aspect ratio was calculated based on the ratio of a particles’ length to its width [46,47].

## 3. Results and Discussion

### 3.1. Fluidity

**Figure 4:**
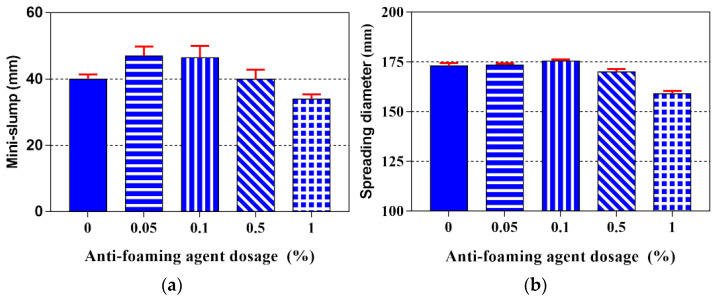
Influence of AFA dosage on the (**a**) mini-slump and (**b**) spreading diameter of 3DPC.

The 3D printing of cementitious materials is an extrusion-based process. Thus, fresh 3DPC should have a favorable fluidity at the pumping and extrusion stage [48,49,50,51]. The influence of various dosages of AFA on mini-slump and spreading diameter is shown in Figure 4a,b, respectively. It can be seen from this figure that the mini-slump and spreading diameter of all of the samples was approximately 40 mm and 170 mm, respectively. Preliminary test results showed that all samples had appropriate workability that could be continuously extruded from the printing nozzle. As seen in Figure 4a, the mini-slump initially increases up to an AFA content of 0.05% and then decreases with higher contents of AFA. Increasing the contents of AFA from 0 to 0.05% resulted in about a 17.5% enhancement of the mini-slump, while increasing the contents of AFA from 0.05% to 1.0% resulted in a 1.05% reduction of the mini-slump. It can be seen from Figure 4b that the 3DPC spreading diameter exhibits similar behavior to that of the mini-slump, which also shows a parabolic tendency. Compared to that of the control sample (AFA0), mixtures containing 0.05% and 0.1% AFA increased the spreading diameter by 0.3% and 1.4%, respectively, while mixtures containing 0.5% and 1.0% AFA decreased the spreading diameter by 1.7% and 8.1%. Therefore, the optimal AFA content is about 0.05%, as it achieved a favorable spreading diameter of fresh 3DPC. It can be noted that the optimal AFA content enables the fresh 3DPC to achieve the desired fluidity.

### 3.2. Evolution of Yield Stress

**Figure 5:**
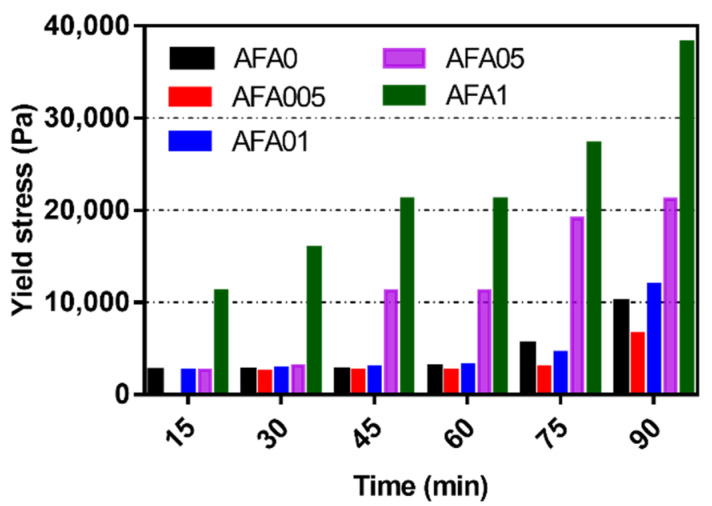
Influence of AFA dosage on the yield stress of 3DPC.

The workability of 3DPC is related to its rheological property and, more particularly, to its yield stress. From a rheological perspective, fresh 3DPC must possess a low viscosity while inside the nozzle. However, high yield stress is essential to resist deformation once it is extruded [52]. Therefore, the evolution of yield stress is an essential indicator of the build-up structure of 3DPC [53]. Figure 5 illustrates the evolution of the yield stress of samples from the time of mixing to 90 min. The yield stress of all of the samples increased with time, which could be associated with the hydration of the cement particles. In addition, the yield stress increased with the increased dosage of AFA over the same time interval. For instance, the yield stress of AFA005, AFA01, AFA05, and AFA1 is 2548, 2810, 3133, and 15,924 Pa at 30 min, respectively. Moreover, the yield stress of fresh 3DPC exhibited a parabolic tendency over the same time interval. Compared to the AFA0 (reference mixture), AFA005 demonstrated a lower yield stress value at all times. However, the yield stress value of AFA01 was always higher than that of the reference mixture except for at 75 min. For instance, increasing the content of AFA from 0 to 0.05% resulted in about an 11.0% reduction of the yield stress at 60 min, while increasing the content of AFA from 0.05% to 0.1% resulted in a 21.7% enhancement of the yield stress at 60 min. The addition of more than 0.5% AFA significantly increased the yield stress of the samples. In the case of AFA05 and AFA1, there was a sudden increase in yield stress after 45 min and 15 min, respectively. However, the excessive dosage increased the stiffness of fresh state 3DPC sharply, which may result in poor extrudability. The evolution of yield stress can be ascribed to the development of the cement paste structure, which is dominated by cement hydration products, such as C-S-H and CH. The components in AFA may alter the hydration behavior of the cement, resulting in the variation in the yield stress of fresh 3DPC.

### 3.3. Strength

**Figure 6:**
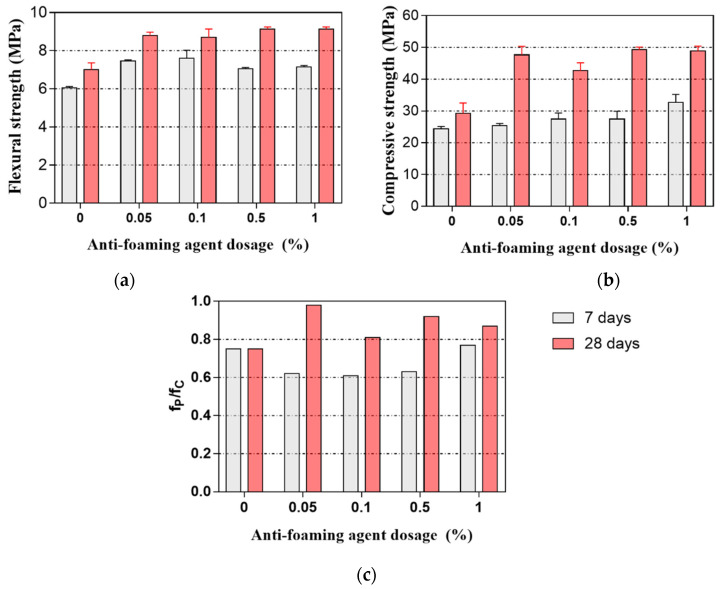
Strength development of 3DPC. (**a**) Flexural strength, (**b**) compressive strength, and (**c**) f_P_/f_C_, where f_P_ and f_C_ represent the compressive strength of the 3D printed mortar and the compressive strength of the mould cast mortar.

Figure 6a,b illustrate the flexural and compressive strength of 3DPC at 7 and 28 days. As expected, adding AFA resulted in higher flexural and compressive strength at 7 and 28 days than that of the control sample without the addition of AFA. The flexural strength of the samples incorporating 0.05–1% AFA achieved 16.5–25.6% and 24.3–30.4% at 7 and 28 days, respectively. The compressive strength of the samples incorporating 0.05–1% AFA achieved 4.1–34.0% and 45.6–67.9% at 7 and 28 days, respectively. These results indicate that AFA can significantly improve the flexural and compressive strength of 3DPC, which can be attributed to the decrease of the air-void content, as discussed later. It was worth noting that the flexural strength of 3DPC at 28 days initially increased to an AFA content of 0.05% and then increased slowly with higher contents of AFA. The compressive strength of 3DPC at 28 days also exhibited similar behavior to that of the flexural strength. Hence, the optimal amount of AFA in 3DPC is about 0.05%, at which the mini-slump and spreading diameter of the designed 3DPC can be the largest, as shown in Figure 4.

Figure 6c shows the compressive strength ratio of 3D printed mortar and mould cast mortar at 7 and 28 days. It can be seen from this figure that all samples at 28 days exhibited a higher compressive strength ratio than that at 7 days. Furthermore, adding 0.05–1% AFA significantly improved the compressive strength ratio at 28 days, where about an 8–30.7% improvement was observed. Among AFA contents, the 0.05% AFA exhibited the highest compressive strength ratio at 28 days. From the results obtained in this work, it is apparent that adding AFA is effective in compensating for the low compressive strength of 3DPC. Additionally, the compressive strength variation between 3D printed samples and mould cast samples can be explained by the interface between the 3D printed layers. The large air-voids at the interface reduce the interface bond area of the 3D printed layers, leading to the reduction of the mechanical strength of the 3DPC.

### 3.4. Air-Void Content

**Figure 7:**
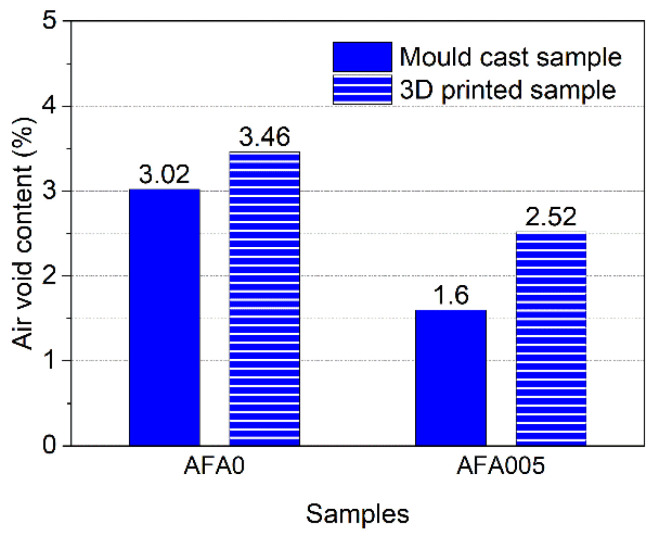
Air-void content of 3DPC at 28 days.

Figure 7 shows the air-void content in samples incorporating 0 and 0.05% AFA at 28 days. As expected, both the mould cast samples and the 3D printed samples incorporating 0.05% AFA exhibited lower air-void contents than those without the addition of AFA. When 0.05% AFA was added, the mould cast sample and the 3D printed sample reduced the air-void content by 47.1% and 27.2%, respectively. The higher strength of the cementitious materials can be achieved by reducing air bubbles [54,55,56]. Therefore, increasing the AFA content from 0 to 0.05% densifies the microstructure of the cementitious materials and significantly improves the flexural and compressive strength. In addition, the air-void content in the 3D printed samples were higher than those of the mould cast sample counterparts. For instance, the air-void in the 3D printed sample with 0.05% AFA was 57.5% higher than that of the mould cast samples with 0.05% AFA. This can be attributed to the layer-by-layer 3DPC construction process without vibration. The increased air-void results in the decrease of the compactness of the 3DPC, therefore causing lower strength, as shown in Figure 6c.

### 3.5. Air-Void Size Distribution

**Figure 8:**
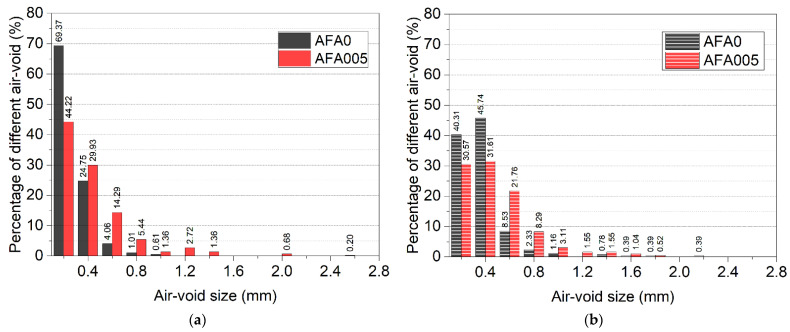
Air-void size distribution of mortar at 28 days. (**a**) Mould cast samples, (**b**) 3D printed samples.

The air-void distribution of the mould cast samples and 3D printed samples at 28 days, observed by electron microscopy with a magnification of ×120, is shown in Figure 8a,b. The relationship between pore volume and pore diameter in the range of 0.1–2.6 mm was determined. These air-voids affect the strength and durability of cementitious materials because of their large size. It can be seen from this figure that the air-voids with a diameter larger than 2.0 mm both in the mould cast samples and the 3D printed samples were eliminated by the addition of 0.05% AFA, indicating that the presence of AFA is beneficial for air-void modification. The largest air-void size was reduced from 2.2 mm to 1.8 mm in the 3D printed mortar and from 2.6 mm to 2.0 mm in the mould cast mortar. AFA decreased the coarse air-voids with a diameter bigger than 2.0 mm in the hardened cementitious materials, which played a positive role in optimizing the air-void structure and in improving mechanical strength. These results confirm a dense structure of the cementitious materials containing 0.05% AFA, which agrees with the air-void content analyses. In addition, the results showed that the air-voids with a particle size of 0.1–0.4 mm in the 3D printed samples with 0.05% AFA were decreased, while air-voids larger than 0.4 mm were increased. A similar phenomenon can also be observed in the mould cast mortars. This may be due to the fact that some of the air-voids smaller than 0.4 mm in the 3D printed cementitious materials have been broken down into smaller air-voids that cannot be observed by the electron microscope at this magnification. The active components in AFA replace surfactant molecules of air bubbles, thus destabilizing the lamella wall built on surfactant, resulting in the fracture of bubbles [57,58]. The AFA mechanism also indicates that the large bubbles in 3DPC decompose into small bubbles. Air-voids smaller than 0.1 mm will be further investigated by backscattered electron image analysis in the future.

### 3.6. Electron Microscope Analysis

**Figure 9:**
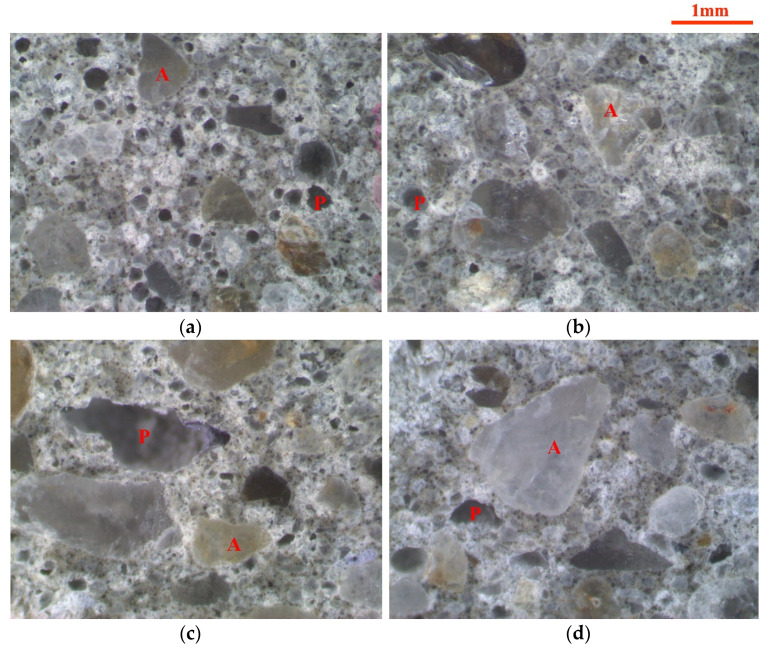
Typical electron microscope images of mould cast mortar and 3D printed mortar. (**a**) Mould cast sample without AFA, (**b**) mould cast sample with 0.05% AFA, (**c**) 3D printed sample without AFA, and (**d**) 3D printed sample with 0.05% AFA, where in the images, A and P represent fine aggregate and the air-void, respectively.

The electron microscope observations on the mould cast mortar and 3D printed mortar with and without the addition of AFA were conducted to examine the pore morphology. Typical electron microscope images of the 3D printed mortar and the mould cast mortar are shown in Figure 9. The air-void and fine aggregate in the images can be identified. In the images, A and P represent the fine aggregate and the air-void, respectively. Figure 9c shows the large air-voids along with the layer orientation in the printed samples, and this type of air-void is minimized by the addition of AFA, as shown in Figure 9d. The presence of large air-voids can be explained by the layer-by-layer deposition process of 3DPC. It is clearly seen that the mould cast sample with 0.05% AFA has very few air-voids than that of the mould cast sample without AFA, which indicates that the mould cast mortar modified by AFA is denser than that of the control mortar without AFA. A similar phenomenon can also be observed in 3D printed mortars. Therefore, the use of AFA leads to a compact 3D printed mortar with a significant reduction of air-voids.

The electron microscope images also represent the air-void morphology. It can be observed that the change in air-void morphology was significant between the 3D printed samples and the mould cast samples. The air-voids in the mould cast samples were mostly elliptical, while the air-voids in the 3D printed samples were mostly stripped with sharp edges. Peng et al. [59] also found parallel strips in 3DPC through microscopic observation, leading to an orthotropic behavior. Sharp edges could increase stress concentration and could then decrease the mechanical stress of the cementitious materials [60]. This is one of the main reasons why the strength of 3D printed samples is lower than that of mould cast samples.

**Figure 10:**
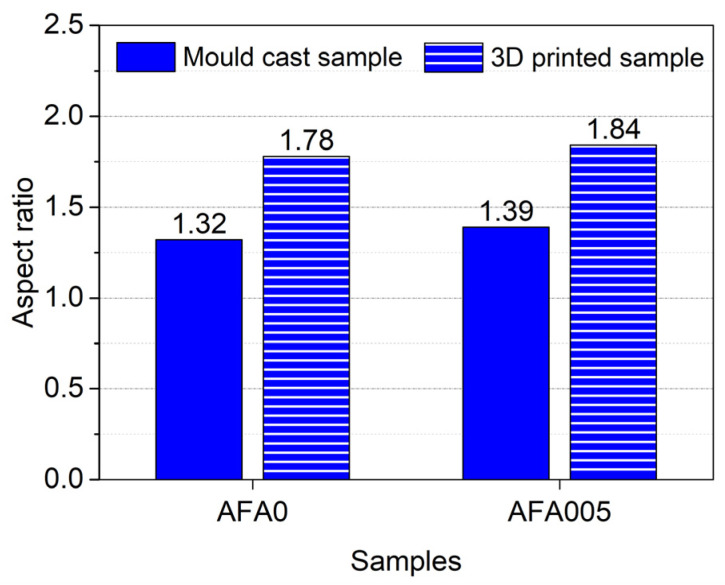
Aspect ratio of air-void at 28 days.

Figure 10 shows the aspect ratio of the air-void in cementitious materials with and without AFA at 28 days. It can be seen from the figure that the aspect ratio of the air-void in the samples with 0.05% AFA revealed a slightly higher increase than that of the samples without AFA. Incorporating 0.05% AFA in the 3D printed samples and the mould cast samples led to a 3.4% and 5.3% improvement in the aspect ratio, respectively. Results also showed that the aspect ratio of the air-void in the 3D printed mortar with and without AFA was higher than that of the counterpart mould cast samples. In the 3D printed mortars with and without AFA, the air-void ratio improved by 32.4% and 34.8%, respectively. The increase in the air-void aspect ratio can be attributed to the layer-by-layer deposition process for the 3D printing of cementitious materials. It is noted that increasing the aspect ratio of the air-void will increase the anisotropy of the stress, which means high stress assemblies along the printing direction as the aspect ratio increases. Thus, the compressive strength of the 3D printed mortar is lower than that of the mould cast mortar, as discussed previously. These findings confirm that the morphology of the air-void has substantial influences on the strength of cementitious materials [30,61].

## 4. Conclusions

In this paper, the fluidity, yield stress, mechanical strength, and air-void structure of 3DPC with different AFA dosages were investigated. Based on the obtained results, the following conclusions can be reached:

(1) The mini-slump and spreading diameter of fresh state 3DPC with 0.05% AFA increased by 17.5% and 0.3% compared to samples without AFA, respectively.

(2) The yield stress could be well controlled by the introduction of 0.05–0.1% AFA into fresh state 3DPC.

(3) The flexural strength and compressive strength of the 3D printed samples with 0.05–1% AFA increased by 24.3–30.4% and 45.6–67.9% relative to the 3D printed samples without AFA at 28 days, respectively.

(4) The air-void content of the 3D printed mortar with 0.05% AFA decreased by 47.1% relative to their counterparts without AFA. The sharp reduction of the air-void content leads to a significant increase in the mechanical strength of the 3D printed mortar.

(5) The strength of 3DPC was lower than that of the counterpart mould cast mortar, which can be attributed to its higher air-void content and larger air-void aspect ratio. The compressive ratio of the samples with 0.05% AFA improved by 30.7% compared to the samples without AFA at 28 days. Thus, the addition of AFA is effective in compensating for the low compressive strength of 3DPC.

(6) The air-voids in the 3D printed samples were mostly striped with sharp edges, which induced stress concentration. However, no significant change of air-void morphology was found between the 3D printed samples with and without AFA.

## Figures and Tables

**Table 1:** Chemical composition of Portland cement (% by mass).

SiO_2_	Al_2_O_3_	Fe_2_O_3_	CaO	MgO	SO_3_	CO_2_	Na_2_O	K_2_O	TiO_2_	P_2_O_5_	Others
18.46	4.29	3.50	64.24	1.74	3.05	2.72	0.24	0.60	0.35	0.25	0.28

**Table 2:** Mix proportions of 3D printing cement mortar.

Mixture	PC (%)	LS (%)	NC (%)	AFA (%)	HRWRA (%)	HPMC (%)	Water to Binder Ratio	Binder to Sand Ratio
AFA0	90	10	1	0	0.06	0.1	0.40	0.50
AFA005	90	10	1	0.05	0.06	0.1	0.40	0.50
AFA01	90	10	1	0.1	0.06	0.1	0.40	0.50
AFA05	90	10	1	0.5	0.06	0.1	0.40	0.50
AFA1	90	10	1	1.0	0.06	0.1	0.40	0.50

## Data Availability

Not applicable.
